# The Effect of Head-to-Head Competition on Behavioural Thermoregulation, Thermophysiological Strain and Performance During Exercise in the Heat

**DOI:** 10.1007/s40279-017-0816-x

**Published:** 2017-11-17

**Authors:** Jo Corbett, Danny K. White, Martin J. Barwood, Christopher R. D. Wagstaff, Michael J. Tipton, Terry McMorris, Joseph T. Costello

**Affiliations:** 1 Extreme Environments Laboratory, Department of Sport and Exercise Science, University of Portsmouth, Spinnaker Building, Cambridge Road, Portsmouth, PO1 2ER UK; 2 Department of Sport, Health and Nutrition, Leeds Trinity University, Brownberrie Lane, Horsforth, LS18 5HD UK; 3 Institute of Sport, University of Chichester, College Lane, Chichester, PO19 9PE UK

## Abstract

**Background:**

It has been suggested that pacing is a thermoregulatory behaviour. We investigated the effect of competition on pacing, performance and thermophysiological strain during exercise in the heat and the psychological factors mediating competition effects.

**Method:**

Eighteen males (maximum oxygen uptake [*V* *O*_2max_] 3.69 [0.44] L min^−1^) undertook a preliminary 20-km cool (wet-bulb globe temperature [WBGT] 12 °C) cycling time trial (TT) and three experimental 20-km trials (balanced order): (i) cool TT (CoolSolo); (ii) hot (WBGT 26 °C) TT (HotSolo); (iii) hot head-to-head competition (HotH2H). During TTs, an avatar of the participant’s performance was visible. During HotH2H, participants believed they were competing against another participant, but the competitor’s avatar replicated their own preliminary (cool) TT.

**Results:**

TTs (min:sec [SD]) slowed with increased ambient temperature [CoolSolo 35:31 (2:11) versus HotSolo 36:10 (2:26); *p* = 0.011]. This effect was negated by competition; performances were not different between HotH2H [35:17 (1:52)] and CoolSolo (*p* = 0.160) and were quicker in HotH2H versus HotSolo (*p* = 0.001). End-exercise rectal temperature, mean body temperature and physiological strain index were (*p* < 0.05) higher in HotH2H than either solo condition. Despite faster performance and greater thermophysiological strain, rating of perceived exertion (RPE), thermal comfort and sensation, and perceptual strain index were not different between HotH2H and HotSolo. The difference in end-exercise rectal temperature between HotH2H and HotSolo was related to pre-exercise anticipatory heart rate response (*r* = 0.608, *p* = 0.010) and participants’ propensity for deliberate risk-taking (*B* = 0.12, *p* < 0.001), whereas self-reported resilience predicted change in performance times between HotH2H versus HotSolo (*B* = − 9.40, *p* = 0.010).

**Conclusion:**

Competition changes the relationship between *perceived* and *actual* thermophysiological state, altering behavioural thermoregulation and increasing thermophysiological strain; this could increase heat-illness risk. Psychophysiological and psychological measures may identify susceptible individuals.

## Key Points

**Table:** 

During solo exercise in the heat, participants alter their pacing relative to cooler exercise, reducing work rate, thereby regulating the degree of thermophysiological strain experienced.
During head-to-head competition, this thermoregulatory behaviour is altered in a manner which increases work rate, thereby increasing thermophysiological strain.
The increased thermophysiological strain with head-to-head competition in the heat is not well sensed and indicates that in competitive situations in the heat there may be dissociation between *perceived* and *actual* thermophysiological state.
Some relatively simple psychophysiological and psychological measures may have utility in identifying individuals susceptible to altering their thermoregulatory behaviour during head-to-head competition in the heat.

## Introduction

Prolonged exercise (≥ 30 min) is impaired in hot environments (air temperature ≥ 30 °C) compared with cooler conditions (air temperature ≤ 20 °C) [1], although the effect of ambient temperature on prolonged exercise performance is not dichotomous, but is instead a continuum, with the fastest performances often achieved at a temperature of ~ 10 °C and an exponential slowing occurring as temperature increases beyond this optimum [2, 3]. Early studies using fixed-intensity, time-to-exhaustion models emphasised the role of a ‘critical’ (~ 40 °C) core temperature (*T*_C_) in the aetiology of fatigue in the heat [4]. However, this ‘critical’ threshold has subsequently been challenged, with Ely et al. [5] demonstrating no difference in running velocity during a self-paced 8000-m run in the heat for the portion of the run when *T*_C_ was > 40 °C, compared with the portion of the trial where *T*_C_ was < 40 °C. Nevertheless, overall completion times were slower in the heat compared with when the run was undertaken in cool conditions [5]; during self-paced exercise in a hot environment, performance may be impaired with modest hyperthermia [6] and work rate is often reduced before a ‘critical’ *T*_C_ [7]. The mechanisms underpinning this effect are complex, resulting from an interplay of cardiovascular, peripheral (muscular), central nervous [8] and psychological factors [9]. Nevertheless, voluntary reductions in work rate are, at least in part, intentionally mediated [10] and result in lower metabolic heat production, reduced physiological strain and improved thermal compensability [11]. Thus, it has been suggested that pacing is a thermoregulatory behaviour for preventing excessive body-heat storage [10].

In laboratory studies in the heat, participants typically exercise alone, yet, as has been noted [8], the *T*_C_ recorded in competitive, non-laboratory situations often exceeds that typical during self-paced laboratory trials [1]. Indeed, ‘competition’ has been cited as a risk factor for exertional heat illness [8, 12]. Although laboratory evidence for this assertion is limited, heat-related collapse in athletic competition is well documented in the field [13, 14]. Laboratory studies in cooler environments have shown improved 2000-m cycling performance when athletes believed they were competing against another participant in a simulated race, but were actually competing against an avatar of their own solo performance [15]. Similarly, participants who believed they were ‘racing’ against a previous 4000-m cycling time trial (TT), but were actually racing against an avatar with a 2% higher power, matched the superior performance [16]. If pacing is a thermoregulatory behaviour, it is important to understand the effect of competition on pacing, performance and thermoregulation during exercise in hot conditions.

Understanding of the cognitive basis for the ergogenic effect of competition is evolving. The presence of a competitor reduces the rating of perceived exertion (RPE), possibly by reducing internal attentional focus [17]; RPE is regarded as the key psychophysiological cue for regulating work rate [18]. Presently, it is unclear if thermal sensation (TS) and thermal comfort (TC) are also influenced by attentional focus, although the extent to which they modulate pacing may depend on the magnitude of hyperthermia [10]. Emotions may also be important [19]. Renfree et al. [20] demonstrated that although RPE did not differ between ‘slow’ and ‘fast’ trials, fast trials had high levels of positive affect and low negative affect. Slovic et al. [21] suggested that affectivity and decision making are influenced by the perceived risks and benefits associated with a given behaviour and low-risk perception is associated with a faster initial exercise pace than that adopted by high-risk perceivers [22]. Beyond the influence of affectivity and risk perception, within high-risk sports where severe injury or death is possible, some individuals may purposefully increase exposure to danger by undertaking deliberate risk-taking (DRT) behaviours, or conversely, precautionary behaviours (PB) which minimise and control dangers [23]; the extent to which these behaviours are relevant for competitive performance in a hot environment, where the dangers may be less obvious, remains to be established. The possibility that individuals with certain trait-like characteristics might be more susceptible to the effect of competition is consistent with studies of the placebo effect, whereby individuals scoring high on resilience, altruism and straightforwardness, and low on angry hostility, were more susceptible to a placebo intervention [24].

Accordingly, we tested the following hypotheses. Firstly, solo exercise performance will be reduced in hot versus cool conditions (H_1_). Secondly, head-to-head competition will influence pacing during exercise in the heat resulting in faster performance than during solo exercise in the heat (H_2_). Thirdly, any performance improvement with head-to-head competition will increase thermophysiological strain (H_3_). Finally, performance improvements with head-to-head competition will be related to certain psychological trait-like characteristics (e.g. risk-taking behaviour), or states (e.g. positive affect) (H_4_).

## Method

### Participants

The Institutional Research Ethics Board approved the experimental protocol, which was in accordance with the Helsinki Declaration. Volunteers provided written informed consent and completed a health history questionnaire before participating. Sample size was calculated using G*Power software, assuming a between-groups difference of 0.25 °C and pooled standard deviation of 0.30 °C for our primary outcome measure [rectal temperature (*T*_re_)], with a power of *β* = 0.80 and *α* = 0.05. This indicated a minimum of 15 participants was required. To ensure a balanced study design for three experimental conditions, 18 male, performance level 2 [25] cyclists (mean [SD] age, body mass, height, absolute maximum oxygen uptake [$\dot{V}$ *O*_2max_], relative $\dot{V}$ *O*_2max_, peak power output: 22 [6] years, 76.4 [10.1] kg, 1.80 [0.07] m, 3.69 [0.44] L min^−1^, 48.5 [4.5] mL kg^−1^ min^−1^, 357 [38] W, respectively) were recruited. All participants undertook regular exercise training (≥ 30 min, ≥ 2 × week), abstained from strenuous exercise for 48 h and caffeine and alcohol for 24 h prior to trials and were instructed to consume the same diet (as near as possible) before each trial and to arrive well hydrated, with a further 250 mL of water provide on arrival at the laboratory.

### Design

A within-participant, balanced, crossover design was employed, with participants randomly allocated to a prescribed trial order. In total, participants visited the laboratory on five occasions, separated by ≥ 48 h. On the first attendance they completed questionnaires to measure trait-like psychological characteristics before undertaking an incremental exercise test, followed 30 min later by a 20-km familiarisation solo cycling TT on a computer-generated ‘virtual’ racecourse, in cool [target wet-bulb globe temperature (WBGT) 12 °C (target dry bulb temperature (*T*_db_) 15 °C; target relative humidity (RH) 55%)] conditions. On the second attendance they undertook a further preliminary solo 20-km TT in cool conditions. On the three subsequent attendances, participants undertook the experimental trials (balanced crossover order), consisting of (i) 20-km solo TT, cool conditions (CoolSolo); (ii) 20-km solo TT, hot [target WBGT 26 °C (target *T*_db_ 30 °C; target RH 55%)] conditions (HotSolo); (iii) 20-km head-to-head competition, hot conditions (HotH2H). The HotH2H trial included a deception element, described subsequently in Sect. 2.3.4. A WBGT of 12 °C is classed as a cool environment; a WBGT of 26 °C is moderately hot with a high risk of exertion heat illness for unacclimated individuals undertaking continuous activity [10].

### Protocol

Exercise was undertaken in a 70-m^3^ temperature and humidity controlled chamber (Crowther and Shaw, Huddersfield, UK) with air flow at ~ 2.7 m s^−1^ (Meterman TMA10, Wavetek, San Diego, USA).

#### Trait-Like Psychological Characteristics

Resilience was evaluated using the 10-item CD-RISC questionnaire [26]. Item responses were summed to provide a global resilience score. Good internal consistency (Cronbach’s Alpha) of *ρ* = 0.84 for the CD-RISC was observed. The risk-taking inventory [23] measured risk-taking attitudes and comprised seven items across two orthogonal factors: DRT (e.g. “I deliberately put myself in danger”) and PB (e.g. “I take time to check for potential hazards”). Woodman et al. [23] reported composite reliability scores of 0.64–0.78 for DRT and 0.64–0.71 for PB. Good internal consistency (Cronbach’s Alpha) for DRT (*ρ* = 0.82) and PB (*ρ* = 0.90) subscales was observed in the present study.

#### Incremental Exercise

The incremental test was undertaken in cool conditions (target *T*_db_ 15 °C; target RH 55%). Participants cycled at 60 W (Velotron Dynafit Pro, RacerMate Inc., Seattle, WA, USA) and external work rate increased by 25 W min^−1^ until volitional exhaustion.

#### Time Trials

TT procedures were identical, with the exception of the ambient conditions. Upon arrival, participants were informed which trial they would be undertaking and to complete the ‘virtual’ racecourse (Velotron 3D software, RacerMate Inc., Seattle, WA, USA) as quickly as possible. Subsequently, they completed the measure of subjective fatigue [27] and the 20-item Positive and Negative Affect Schedule (PANAS) to assess mood [28]. The PANAS has been widely used within non-clinical samples and particularly sport samples, and is regarded as a highly reliable measure for such populations, with validation studies indicating that the measure demonstrates excellent construct validity [29]. In addition to its common use and validation, the PANAS also represents a shorter option to alternative measures. Thereafter, following instrumentation, baseline measures, and a 5-minute warm-up on the cycle ergometer at 100 W, participants rested for 5 min and the purpose of the trial was reiterated. They then completed a self-paced ‘all-out’ 20-km TT. During TTs, an avatar representing the participant on the racecourse was visible; distance was displayed but other feedback was occluded.

#### Head-to-Head Competition

Procedures for HotH2H were identical to TTs with the following exceptions. Upon arrival participants were informed they would be competing over the same 20-km racecourse against another participant of similar ability, who would be exercising on an adjacent Velotron ergometer, and that they should try and beat the other competitor. The participants were also informed that they would not be allowed to see the other competitor at any point prior to, during, or after the test in order to minimize possible confounding effects from perceptual cues and inter-personal rivalries. Thus, the participants were kept in separate rooms prior to the exercise test, while during the test the cycle ergometers were separated by screens and participants were instructed that verbal communication was not permitted. Thereafter, they completed questionnaires for measuring subjective fatigue and mood. During the trial, avatars of the participant and the ‘competitor’ were generated on the ‘virtual’ racecourse as previously described [15]. However, whilst participants believed they were competing against another participant, the ‘competitor’ avatar was generated by the software, which replicated the participant’s preliminary (second visit) performance under cool conditions. Participants were unaware of the deception (confirmed via interview post-experiment); the sham competitor was a member of the experimental team who exercised behind the separation screen.

### Measurements

Clothed and naked mass were measured (I-10, Ohaus, NJ, USA) before and after exercise. A wet-bulb globe thermometer measured laboratory conditions (Edale Instruments, Cambridge, UK). Rectal temperature (*T*_re_) was measured by a thermistor at a depth of 15 cm (Edale Instruments, Cambridge, UK) and skin temperature (chest, upper arm, thigh, calf) was measured using thermistors (Edale Instruments, Cambridge, UK), both were logged (Squirrel 2040, Grant Instruments, Cambridge, UK). Mean skin temperature ($\overline{T}_{sk}$) was calculated using Ramanathan [30] with mean body temperature ($\overline{T}_{b}$) calculated as (0.9 × *T*_re_) + (0.1 ×$\overline{T}_{sk}$) [31]. For safety, trials were terminated if *T*_re_ was ≥ 40 °C, or if, upon reaching a *T*_re_ of 39.5 °C, the rate of rise was > 0.15 °C in a 5-minute period or heart rate was within 5 b.min^−1^ of maximum. Expired gases were measured breath-by-breath (Quark CPET, Cosmed, Rome, Italy) and interpolated to 1-sec values with metabolic heat production calculated according to ISO 8996 [32]. Heart rate was measured before and during exercise using an RS800 monitor (Polar electro, Oy, Finland). The average heart rate in the 3 min prior to commencing the 20-km trials was used to provide a psychophysiological index of pre-exercise anxiety. TC, TS [33] and RPE [34] were recorded before trials and at 4-km intervals. Scales were accompanied by standardised instructions and the memory anchoring procedure [35] was used; the preliminary incremental exercise test and 20-km TT also assisted in familiarising participants with the perceptual scales. Physiological Strain Index (PSI) [36] and Perceptual Strain Index (PeSI) [37] were calculated.

### Statistical Analysis

Data are mean (SD) unless stated, with significance as *p* < 0.05. Analysis was performed using IBM SPSS statistics 22. Between-conditions differences in average power, completion times, sweat loss, ambient conditions and psychological state were analysed by one-way repeated measures ANOVA with post-hoc analysis using the least significant difference (LSD) method. Main and Interaction effects for pacing, thermal and cardiovascular variables, TC, TS and PSI were examined by two-way repeated measures ANOVA (Condition × Distance). Effect sizes are reported as partial *η* ^2^ and significant Condition and Interaction effects were investigated using the LSD method. Where sphericity was violated the Greenhouse-Geisser statistic was used. Between-conditions differences in ordinal data (RPE, PeSI) were analysed by Friedman’s test, with post-hoc analysis by Wilcoxon sign-rank tests. Relationships between physiological variables and change in performance time, average power, and end-exercise *T*_re_ between HotSolo and HotH2H were assessed by Pearson’s correlation. Relationships between trait-like psychological variables and change in performance time and end-exercise *T*_re_ between HotSolo and HotH2H were assessed using multiple regression analysis.

## Results

WBGT did not differ (*p* = 0.646) between HotSolo [26.0 °C (0.4)] and HotH2H [26.1 °C (0.4)], but was lower in CoolSolo [11.8 °C (0.4), both *p* < 0.001]. Seventeen participants completed all trials. One participant was withdrawn from two trials (HotSolo and HotH2H) due to the *T*_re_ withdrawal criteria; in each instance they were nearing completion (> 18.75 km covered). Data from beyond 18.75 km in the other trials were excluded for this participant and thermophysiological data at this distance were taken as the terminal data for this individual given the close proximity to the trial end; terminal perceptual data were not available. Analyses were repeated with this participant excluded and were not different from those reported.

### Performance

**Fig. 1:**
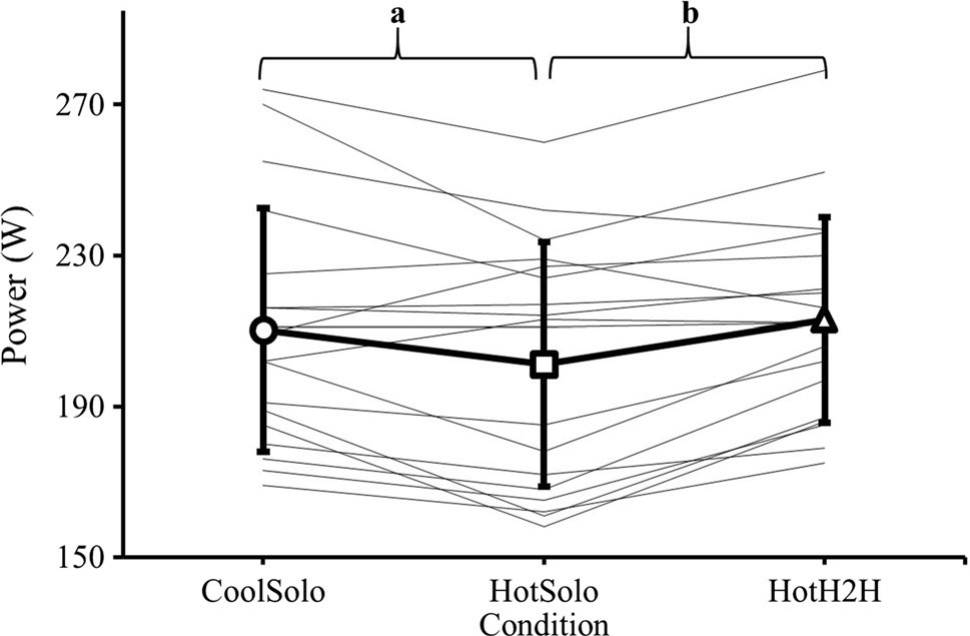
Mean (SD) [thick black line (*n* = 18)] and individual (thin black lines) average power outputs for 20-km time trials in cool (CoolSolo) and hot (HotSolo) environments, and a 20-km simulated head-to-head competition in a hot environment (HotH2H). **a** Significant difference (*p* < 0.05) between CoolSolo and HotSolo; **b** significant difference (*p* < 0.01) between HotSolo and HotH2H

Completion times for CoolSolo, HotSolo and HotH2H were 35:31 (2:11), 36:10 (2:26) and 35:17 (1:52) min:sec (SD), corresponding to average powers of 210 (32), 201 (32) and 213 (27) W, respectively (Fig. 1). Completion time differed between trials (*F*_(2,34)_ = 9.69, *p* < 0.001, partial *η* ^2^ = 0.36); solo TT performance slowed with increased temperature (CoolSolo vs HotSolo, *p* = 0.011), but this was negated by competition (CoolSolo vs HotH2H; *p* = 0.160) and performances were quicker in HotH2H versus HotSolo (*p* = 0.001). Mean power also differed between trials (*F*_(2,34)_ = 8.89, *p* = 0.001, partial *η* ^2^ = 0.34), being reduced with increased temperature during solo exercise (CoolSolo vs HotSolo, *p* = 0.013), but higher in HotH2H versus HotSolo, (*p* = 0.001), and not different between CoolSolo and HotH2H (*p* = 0.323). There was high reliability between CoolSolo performances and the preliminary cool 20-km TT [35:29 (2:15)]; that is, the ‘competitors’ performance, as indicated by a low coefficient of variation (0.9%).

### Pacing

**Fig. 2:**
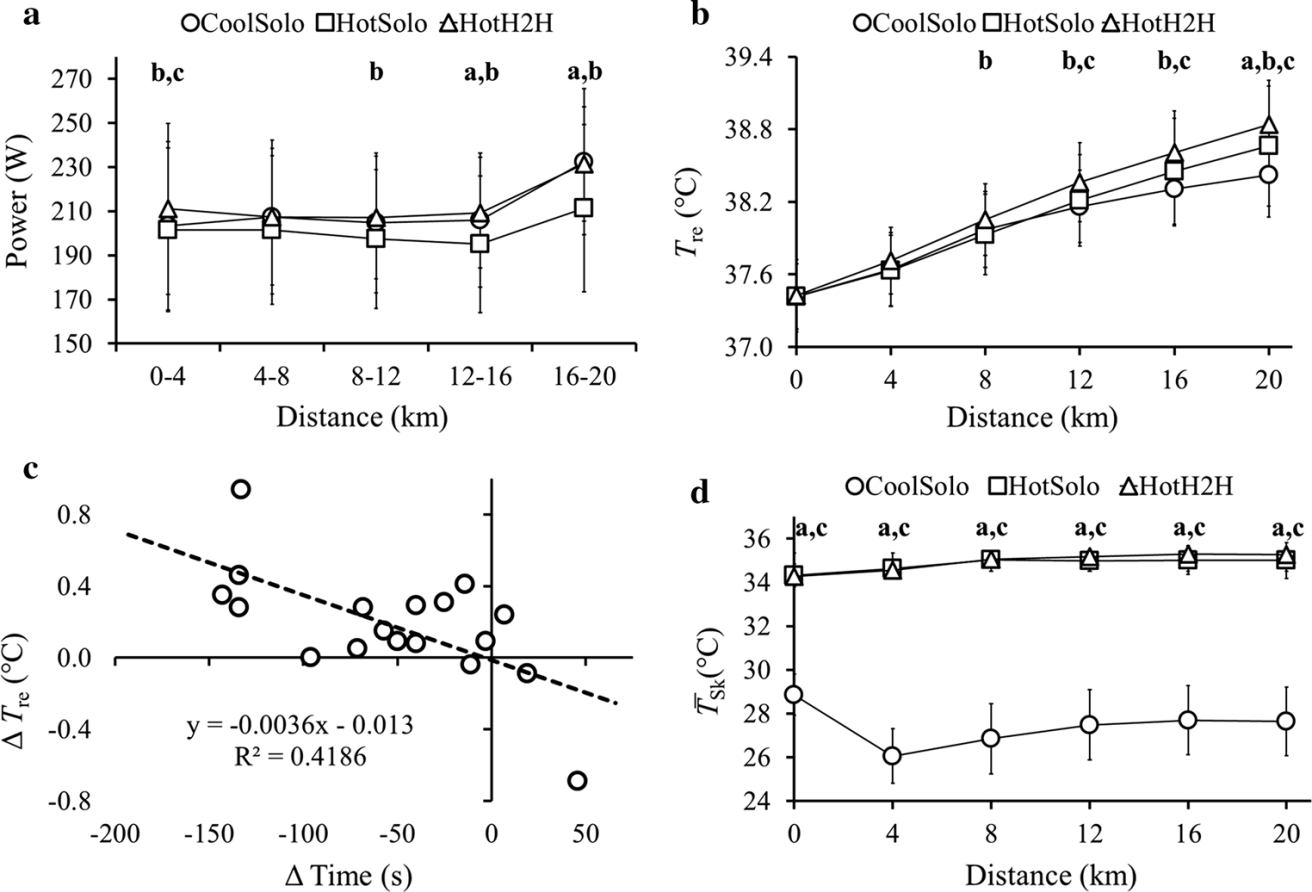
**a** Mean (SD) pacing profile for 4-km segments for 20-km time trials in cool (CoolSolo) and hot (HotSolo) environments, and a 20-km simulated head-to-head competition in a hot environment (HotH2H). **b** Mean (SD) rectal temperature (*T*_*re*_) at 4-km intervals for CoolSolo, HotSolo and HotH2H. **c** Relationship between individual Δ time in HotH2H vs HotSolo and individual Δ end-exercise *T*_re_ in HotH2H vs HotSolo. **d** Mean (SD) mean skin temperature ($\overline{T}_{sk}$) at 4-km intervals for CoolSolo, HotSolo and HotH2H. **a** significant difference (*p* < 0.05) between CoolSolo and HotSolo; **b** significant difference (*p* < 0.05) between HotSolo and HotH2H; **c** significant difference (*p* < 0.05) between CoolSolo vs HotH2H

There were main (Condition *F*_(2,34)_ = 9.35, *p* = 0.001, partial *η* ^2^ = 0.36; Distance *F*_(1.8,31.2)_ = 13.39, *p* < 0.001, partial *η* ^2^ = 0.44) and Interaction (*F*_(3.1,52.8)_ = 3.82, *p* = 0.014, partial *η* ^2^ = 0.18) effects on pacing. A higher initial power (0–4 km) was adopted in HotH2H versus CoolSolo and HotSolo. From 8–12 km until completion, power was higher in HotH2H versus HotSolo. Similarly, from 12–16 km until completion power was higher in CoolSolo versus HotSolo (Fig. 2a). Analysis of CoolSolo versus the preliminary cool 20-km TT (i.e. the ‘competitor’) showed that power changed over Distance (*F*_(2.2,38.1)_ = 24.25, *p* < 0.001, partial *η* ^2^ = 0.59), but to the same extent in each condition (Condition *p* = 0.854, Interaction *p* = 0.768), indicating similar pacing.

### Thermal Responses

Main (Condition *F*_(2,34)_ = 4.53, *p* = 0.018, partial *η* ^2^ = 0.21; Distance *F*_(1.2,21.0)_ = 230.09, *p* < 0.001, partial *η* ^2^ = 0.93) and Interaction (*F*_(3.3,55.6)_ = 7.88, *p* < 0.001, partial *η* ^2^ = 0.32) effects were evident on *T*_re_. *T*_re_ did not differ between conditions before commencing trials, but from 8 km onwards, *T*_re_ was higher in HotH2H versus HotSolo and higher in HotH2H versus CoolSolo from 12 km onwards. *T*_re_ was higher in HotSolo versus CoolSolo at 20 km only (Fig. 2b). The change in end-exercise *T*_re_ between HotH2H and HotSolo was related to the change in performance times between these conditions [*r* = −0.647, *p* = 0.004 (Fig. 2c)], as well as the change in power between the conditions in absolute [i.e. W (*r* = 0.639, *p* = 0.004)] and relative [i.e. W kg^−1^ (*r* = 0.638, *p* = 0.004)] terms. Main (Condition *F*_(1.2,20.2)_ = 524.83, *p* < 0.001, partial *η* ^2^ = 0.97; Distance *F*_(2.1,36.5)_ = 42.40, *p* < 0.001, partial *η* ^2^ = 0.71) and Interaction (*F*_(2.3,39.3)_ = 37.07, *p* < 0.001, partial *η* ^2^ = 0.69) effects were evident on $\overline{T}_{sk}$, which was higher in HotH2H and HotSolo versus CoolSolo throughout, but not different between HotH2H and HotSolo (Fig. 2d). Main (Condition *F*_(2,34)_ = 115.31, *p* < 0.001, partial *η* ^2^ = 0.87; Distance *F*_(1.3,21.5)_ = 214.37, *p* < 0.001, partial *η* ^2^ = 0.93) and Interaction (*F*_(3.2,54.4)_ = 13.51, *p* < 0.001, partial *η* ^2^ = 0.44) effects were also evident for $\overline{T}_{b}$ (higher in HotSolo and HotH2H versus CoolSolo throughout, and higher in HotH2H versus HotSolo from 12 km).

Sweat losses (*n* = 17) were 0.87 (0.33), 1.33 (0.30), 1.39 (0.36) L hr^−1^ for CoolSolo, HotSolo and HotH2H, respectively. Sweat loss differed between conditions (*F*_(2,32)_ = 54.39, *p* < 0.001, partial *η* ^2^ = 0.77), being lower in CoolSolo versus HotSolo and HotH2H (both *p* < 0.001), but not different between HotSolo and HotH2H.

### Cardiometabolic Responses

**Fig. 3:**
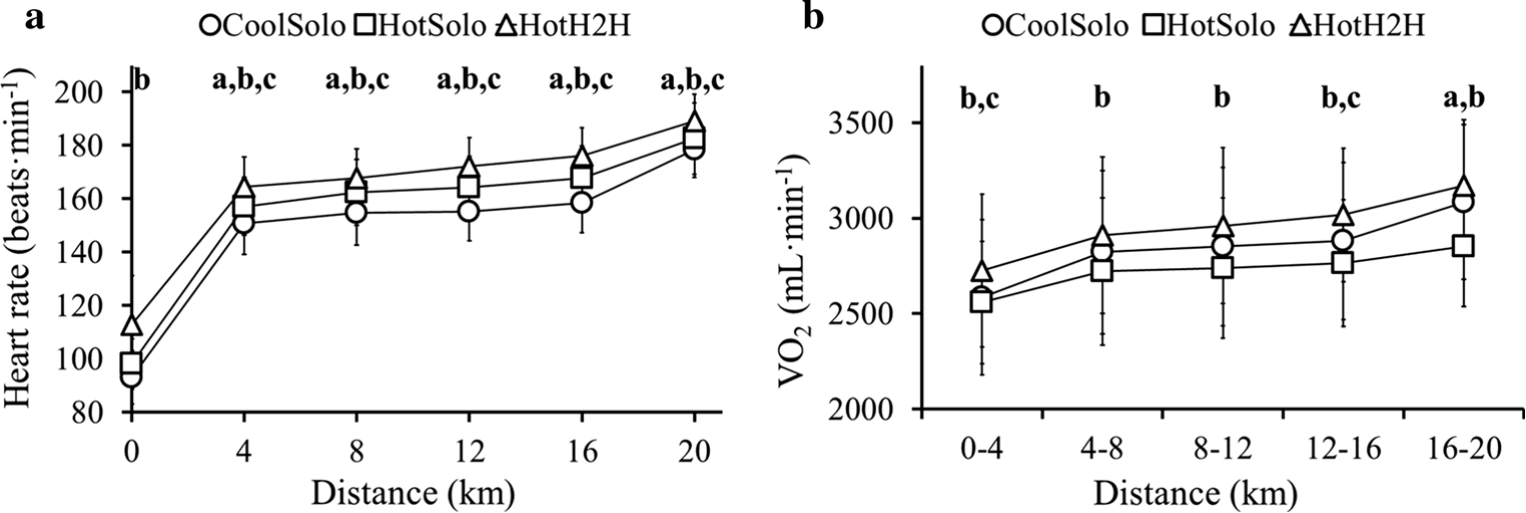
**a** Mean (SD) heart rate at 4-km intervals for 20-km time trials in cool (CoolSolo) and hot (HotSolo) environments, and a 20-km simulated head-to-head competition in a hot environment (HotH2H). **b** Mean (SD) rate of oxygen uptake (*n* = 17) for 4-km segments for CoolSolo, HotSolo and HotH2H. $\dot{V}$ *O*_2max_ maximum oxygen uptake. **a** Significant difference (*p* < 0.05) between CoolSolo and HotSolo; **b** significant difference (*p* < 0.05) between HotSolo and HotH2H; **c** significant difference (*p* < 0.05) between CoolSolo and HotH2H

Main (Condition *F*_*(*2,34)_ = 46.82, *p* < 0.001, partial *η* ^2^ = 0.73; Distance *F*_(2.1,35.0)_ = 385.08, *p* < 0.001, partial *η* ^2^ = 0.96) and Interaction (*F*_(4.2,71.2)_ = 2.78, *p* = 0.031, partial *η* ^2^ = 0.14) effects were evident for heart rate. Upon commencement, heart rate was higher in HotH2H than HotSolo. Thereafter, heart rate differed between all conditions, being highest in HotH2H and lowest in CoolSolo (Fig. 3a). The average heart rate in the 3 min prior to commencing the 20-km trials (used as a psychophysiological index of pre-exercise anxiety) differed between conditions [*p* < 0.001 (*n* = 17)], being highest in HotH2H and lowest in CoolSolo. The individual change in pre-exercise heart rate between HotH2H and HotSolo correlated with the change in end-exercise *T*_re_ between these conditions [*r* = 0.608, *p* = 0.010 (*n* = 17)], but not with the change in performance times or power (absolute or relative).

Main effects were evident for $\dot{V}$ *O*_2_ (*n* = 17, Condition *F*_(2,32)_ = 9.02, *p* = 0.001, partial *η* ^2^ = 0.36; Distance *F*_(1.7,28.0)_ = 30.59, *p* < 0.001, partial *η* ^2^ = 0.66) and metabolic heat production (*n* = 17, Condition *F*_*(*2,32)_ = 10.41, *p* = 0.001, partial *η* ^2^ = 0.39; Distance *F*_(2.0,32.0)_ = 185.06, *p* < 0.001, partial *η* ^2^ = 0.92); the Interaction effect was also significant for $\dot{V}$ *O*_2_ (*F*_*(*3.8,61.6)_ = 2.97, *p* = 0.028, partial *η* ^2^ = 0.16). $\dot{V}$ *O*_2_ (Fig. 3b) and metabolic heat production were reduced in HotSolo versus CoolSolo at 16–20 km and higher in HotH2H versus HotSolo throughout. $\dot{V}$ *O*_2_ and metabolic heat production were also higher in HotH2H versus CoolSolo at 0–4 and 12–16 km, with higher metabolic heat production at 8–12 km.

### Perceptual Responses

**Fig. 4:**
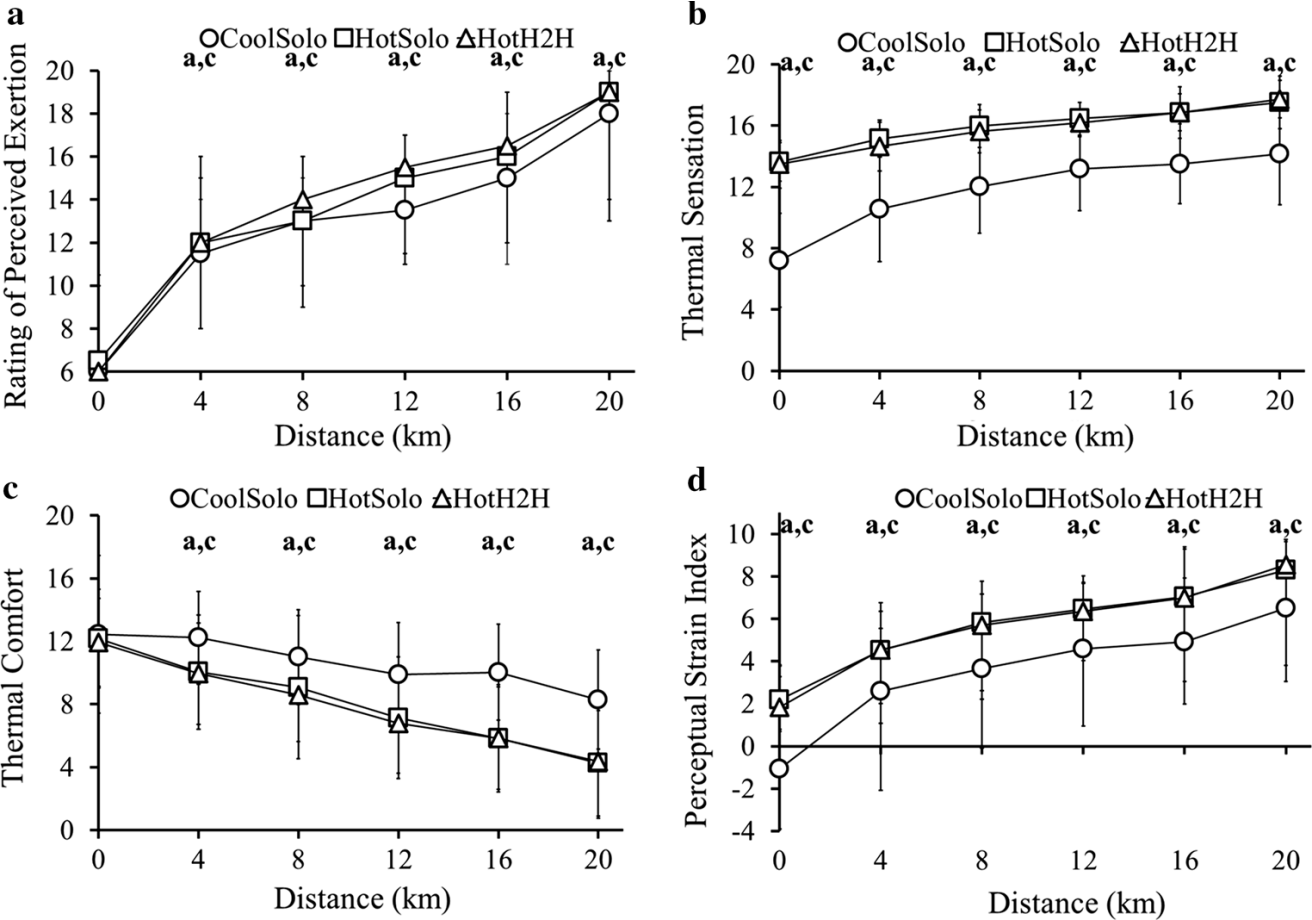
**a** Median (range) rating of perceived exertion at 4-km intervals for 20-km time trials in cool (CoolSolo) and hot (HotSolo) environments and a 20-km simulated head-to-head competition in a hot environment (HotH2H), *n* = 18 for all points except 20 km where *n* = 17. **b** Mean (SD) thermal comfort at 4-km intervals for CoolSolo, HotSolo and HotH2H, *n* = 17. **c** Mean (SD) thermal sensation at 4-km intervals for CoolSolo, HotSolo and HotH2H, *n* = 17. **d** Median (range) Perceptual Strain Index at 4-km intervals for CoolSolo, HotSolo and HotH2H, *n* = 18 for all points except 20 km, where *n* = 17. **a** Significant difference (*p* < 0.05) between CoolSolo and HotSolo; **b** significant difference (*p* < 0.05) between HotSolo and HotH2H; **c** significant difference (*p* < 0.05) between CoolSolo and HotH2H

RPE was not different between conditions at baseline, but thereafter was lower in CoolSolo versus HotSolo and HotH2H, but not different between HotSolo and HotH2H (Fig. 4a). TS (Fig. 4b) and TC (Fig. 4c) differed between Conditions (TS *F*_(1.2,19.5)_ = 50.74, *p* < 0.001, partial *η* ^2^ = 0.76; TC *F*_(2,32)_ = 15.17, *p* < 0.001, partial *η* ^2^ = 0.49), over Distance (TS *F*_(2.3,37.2)_ = 71.12, *p* < 0.001, partial *η* ^2^ = 0.82; TC *F*_(2.1,33.8)_ = 47.81, *p* < 0.001, partial *η* ^2^ = 0.75) and with their Interaction (TS *F*_(3.8,60.5)_ = 6.13, *p* < 0.001, partial *η* ^2^ = 0.28; TC *F*_(3.8,61.4)_ = 3.62, *p* = 0.011, partial *η* ^2^ = 0.18). Participants perceived themselves to be hotter in HotSolo and HotH2H versus CoolSolo from the start and were less comfortable in HotSolo and HotH2H versus CoolSolo from 4 km.

### Strain Indices

Main (Condition *F*_*(*2,34)_ = 22.69, *p* < 0.001, partial *η* ^2^ = 0.57; Distance *F*_(1.7,28.9)_ = 612.69, *p* < 0.001, partial *η* ^2^ = 0.97) and Interaction (*F*_*(*3.7,63.6)_ = 3.45, *p* = 0.015, partial *η* ^2^ = 0.17) effects were evident on PSI, which was higher in HotH2H at each interval; from 12 km onwards, PSI was also higher in HotSolo than CoolSolo. PeSI did not differ between HotSolo and HotH2H at any point, but was lower in CoolSolo (Fig. 4d).

### Psychological Responses

There was no Condition effect on subjective fatigue or PANAS. Multiple regression analyses indicated that resilience, DRT and PB explained 66.8% of the variance in change in performance time between HotSolo and HotH2H (_adj_ *R* ^2^ = 0.66, *p* < 0.001). Of the individual predictors, only resilience was significant [*B* = − 9.40, *p* = 0.010, lower level confidence interval (LLCI) = − 16.16, upper level confidence interval (ULCI) = − 2.66]. Resilience, DRT and PB also explained 69.0% of the variance (_adj_ *R* ^2^ = 0.69, *p* < 0.001) in change in end-exercise *T*_re_ between HotSolo and HotH2H. Post-hoc tests revealed DRT significantly predicted change in end-exercise *T*_re_ between HotSolo and HotH2H (*B* = 0.12, *p* < 0.001, LLCI = 0.05 ULCI = 0.18); resilience and PB were not predictive (Table 1). In line with Woodman et al. [23], we tested the potential interaction of DRT and PB in predicting change in end-exercise *T*_re_ and change in performance time between HotSolo and HotH2H. Factors were standardized before creating an interaction term but this accounted for no further significant proportion of variance in change in end-exercise *T*_re_ or change in performance time between HotSolo and HotH2H.

**Table 1:** Relationship between resilience, precautionary behaviour, deliberate risk taking, change in performance time (HotSolo vs HotH2H) and change in end-exercise *T*_re_ (HotSolo vs HotH2H)

	*B*	SE	β	*T*	LLCI	ULCI
∆ performance time (s)	379.71	79.48		4.78**	209.24	550.18
CD-RISC	− 9.41	3.15	− 0.64	− 2.99*	− 16.16	− 2.66
PB	− 6.89	4.31	− 0.25	− 1.60	− 16.14	2.36
DRT	− 4.30	5.41	− 0.16	− 0.79	− 15.90	7.30
∆ end-exercise *T*_re_ (°C)	− 1.12	0.430		− 0.260	− 2.04	− 0.19
CD-RISC	0.009	0.017	0.104	0.505	− 0.02	0.05
PB	0.014	0.023	0.087	0.580	− 0.03	0.06
DRT	0.117	0.029	0.784	3.982**	0.05	0.18

*CD*-*RISC* Connor–Davidson resilience scale, *DRT* deliberate risk taking, *HotSolo* a 20-km time trial undertaken in a hot environment; *HotH2H* a 20-km simulated head-to-head competition in a hot environment, *LLCI* lower level confidence interval, *PB* precautionary behaviour, *T*_re_ rectal temperature, *ULCI* upper level confidence interval; *p < 0.05, **p < 0.01

## Discussion

This study is the first to demonstrate that head-to-head competition alters behavioural thermoregulation (i.e. pacing) during exercise in the heat. Compared with solo exercise in the heat, head-to-head competition resulted in increased external work rate [+11.8 (12.3) W], metabolic heat production, and thermophysiological strain, and improved performance [−53 (57) s] such that participants matched their (solo) performance under cool conditions. Despite thermophysiological differences, perceptual responses were unchanged with head-to-head competition. Moreover, the increased end-exercise *T*_re_ (+0.18 (0.32) °C) with head-to-head competition was related to the anticipatory heart rate increase, and was predicted by participants’ attitudes towards DRT, whereas self-reported resilience predicted change in performance time between HotSolo and HotH2H. These findings are important because they are consistent with the suggestion that competition is a risk factor for exertional heat illness [8, 12], provide potential mechanistic insight into the underlying processes (disassociation between *perceived* and *actual* thermophysiological state) and demonstrate that simple psychophysiological and psychological measures may have utility in identifying susceptible individuals.

Increased ambient temperature impaired solo exercise, as has been demonstrated previously [1–3]. Thus, H_1_ (solo performance will be reduced in hot versus cool conditions) is accepted. Although initial pacing profiles were similar, power was lower in HotSolo versus CoolSolo from 12 km onwards, which is in accordance with a recent meta-analysis [7]. The reduced power occurred with a relatively modest *T*_re_ (~ 38.0 °C) and is consistent with the assertion that pacing is a thermoregulatory behaviour which prevents excessive body-heat storage in advance of a high *T*_C_ [10]. The driver(s) influencing this process are unclear, although thermophysiological (*T*_re_ [4], skin temperature and cardiovascular strain [38], heat storage [39]), and perceptual (TS, TC, [10], RPE [18]) factors have been implicated. Indeed, differences were evident between HotSolo and CoolSolo in some thermophysiological (increased $\overline{T}_{re}$, $\overline{T}_{sk}$, $\overline{T}_{b}$, $\dot{V}$ *O*_2_, heart rate, PSI) and perceptual indices (increased RPE, TS, PeSI, reduced TC). Although the onset of differences did not always coincide with the altered pacing, this does not preclude their involvement as behavioural cues and certain thresholds may need to be surpassed before influencing behaviour.

Head-to-head competition altered pacing in the heat such that the performance decrement in HotSolo versus CoolSolo was abolished and participants matched their CoolSolo performances. Indeed, 11 out of the 18 participants (61%) were able to record a faster time in HotH2H than in CoolSolo, such was the ergogenic effect of the head-to-head competition. Thus, H_2_ (head-to-head competition will influence pacing during exercise in the heat resulting in faster performance times than during solo exercise in the heat) is accepted. The influence of head-to-head competition was greatest early on (0–4 km), where power was higher in HotH2H than HotSolo *and* CoolSolo; despite the high ambient temperature, participants exceeded their ‘competitor’s’ pace, generated (unknowingly) by their own cool-conditions performance. The early stages of exercise are most susceptible to manipulation because initial pace is primarily generated using feed-forward processes, incorporating knowledge of exercise endpoint, previous experience, environmental conditions, and motivational factors [18], and any afferent feedback cues influencing behaviour are less intense. Our data are similar to pacing in Olympic and World Championship distance-running races, where athletes initially adjust their speed to match their opponents, rather than adopting their usual pace [40]. According to decision-making theory, which has recently been applied to pacing [41], a potentially large payoff, such as beating a competitor, might encourage a riskier strategy and tolerance of greater physiological disruption (or harm). From a neuroscience perspective, increased motivation stimulates dopamine release from the ventral tegmental area and activation of the motivation/reward pathway via the dopaminergic mesolimbic pathway [42]; norepinephrine also plays a role [43]. Indeed, the dopamine/norepinephrine reuptake inhibitor, bupropion, improves exercise in the heat [44]. The release of norepinephrine and epinephrine is also consistent with a psychophysiological stress response, which is in keeping with the anticipatory heart-rate rise [45] in HotH2H, whereas cortisol, which is also released in stressful situations, modulates behaviour related to increased motivated decision making, where high-risk choices yield potentially big rewards [46]. Exogenous cortisol supplementation increases risk-taking behaviour [47], which is associated with a higher initial pace [22]. Although speculative, these responses could account for the faster early pace in HotH2H. Future studies should seek to investigate these putative mechanisms using appropriate neuro-imaging techniques and blood measures, including catecholamines, neurotransmitters and stress hormones.

Beyond the initial exercise period, participants matched the ‘competitor’ performance, such that power was higher in HotH2H than HotSolo (from 8 km) and not different from CoolSolo (from 4 km). Consequently, metabolic heat production increased, leading to increased thermophysiological strain (higher *T*_re_, $\overline{T}_{b}$, heart rate and PSI in HotH2H versus HotSolo) and enabling acceptance of H_3_ (performance improvements with competition will increase thermophysiological strain). The implications of this finding are significant; high body temperatures are associated with exertional heat illnesses and prolonged high body temperatures (*T*_re_ >40 °C) can lead to exertional heat stroke [13]. Thus, our data are consistent with the hypothesis that competition increases heat-illness potential [8, 12]. Importantly, unlike during solo exercise, where thermophysiological differences were paralleled by perceptual differences, there were no between-conditions differences in TS, TC, RPE or PeSI between HotH2H and HotSolo.

It is possible that the magnitude of thermophysiological differences between HotH2H and HotSolo was below that influencing perception, or that key perceptual drivers were unaffected. For example, ${\overline{T}}_{sk}$, which influences TS [10], was not different between these conditions. Alternatively, head-to-head competition may alter the relationship between *perceived* and *actual* thermophysiological state. Previous research has shown some ‘centrally acting’ interventions enable athletes to ignore cues which normally regulate exercise in the heat. Methylphenidate (a dopamine reuptake inhibitor) and bupropion both improved performance and resulted in higher deep body temperatures during exercise at 30 °C, ~ 55% RH [44, 48], whereas psychological skills training enabled participants to run further during 90 min of exercise at 30 °C, 40% RH [9]; in each case there was no change in RPE or perceived thermal stress. Similarly, in temperate conditions, the presence of a competitor reduces internal attentional focus and limits attentional resources directed to afferent sensory feedback, thereby enabling a higher power for a given RPE [17]. Thus, head-to-head competition may have reduced internal attentional focus, limiting attentional resources for afferent sensory feedback and enabling a higher power, cardiovascular and thermal strain for a given perceptual state.

A further important finding was that the heart-rate rise with head-to-head competition was related to the change in *T*_re_ between HotH2H and HotSolo. Similarly, participants’ perceptions of their own resilience influenced their performance in a competition scenario, whereas DRT predicted the increased *T*_re_ with competition. Collectively, these findings enable acceptance of H_4_ (performance improvements with head-to-head competition will be related to certain trait-like and state psychological characteristics). These findings suggest that it may be possible to identify individuals most influenced by head-to-head competition in a hot environment by simple psychological and psychophysiological measures. That is, those with high self-rated resilience, a high propensity for risk-taking behaviour and/or a pronounced anticipatory heart rate response, may be more susceptible to the effects of head-to-head competition.

The present study was not without limitation. For ethical and safety reasons, we could not induce exertional heat illness per se and whilst high deep-body temperatures are associated with exertional heat illness, the aetiology is complex and many individuals achieve high deep-body temperatures without developing heat illness [1]. Moreover, the average terminal *T*_re_ achieved in HotH2H was below that associated with heat stroke [13], although many of our participants (44%) achieved a *T*_re_ ≥ 39 °C and the lag inherent within this measurement site makes it likely that the ‘true’ terminal temperature was somewhat higher, whereas inter-individual variation exists in the deep-body temperatures of individuals with exertional heat illnesses [13]. Finally, given that an ergogenic effect of competition has previously been demonstrated under cooler conditions [15], an ergogenic effect of competition in the heat was perhaps not unexpected. However, the key point is that an increased ambient temperature elicited a behavioural change (altered pacing) during solo exercise that regulated the thermal strain experienced by the participant, but in the competitive situation this behaviour was altered with the consequence that thermal strain was increased. Although further work is required to clarify the extent to which this increases susceptibility to exertional heat illness, recent research demonstrated that group-paced activities, which are similar to the head-to-head competitive situation, account for 70% of exertional heat illness cases and 78.5% of subsequent hospitalizations among UK military personnel, the remainder occurring during self-paced exercise [14].

## Conclusion

Compared with solo exercise in the heat, head-to-head competition in a hot environment alters behavioural thermoregulation (i.e. pacing), resulting in faster performances. Consequently, metabolic heat production and thermophysiological strain are increased, but this is not reflected in perceptual measures. These novel data are consistent with the hypothesis that competition is a risk factor for heat illness. We suggest that this effect may result from neurochemical changes due to a psychophysiological stress response or motivational effects, whereas reduced internal focus might alter the relationship between *perceived* and *actual* thermophysiological state. Finally, individuals with a propensity for DRT, or high levels of resilience, may be more sensitive to the effects of competition, indicating that certain trait-like characteristics might help identify those at increased risk of heat illness.
